# Regulation of Immune Responses and Chronic Inflammation by Fibroblast-Like Synoviocytes

**DOI:** 10.3389/fimmu.2019.01395

**Published:** 2019-06-19

**Authors:** Hiroyuki Yoshitomi

**Affiliations:** Department of Regeneration Science and Engineering, Institute for Frontier Life and Medical Sciences, Kyoto University, Kyoto, Japan

**Keywords:** fibroblast-like synoviocytes (FLSs), synovial tissue, rheumatoid arthritis, non-immune cells, immune cells, autoantibodies, ectopic lymphoid-like structures (ELSs)

## Abstract

Synovial tissue is a membranous non-immune organ lining joint cavities where it supports local immune responses, and functions directly and indirectly in joint destruction due to chronic inflammatory diseases such as rheumatoid arthritis (RA). Fibroblast-like synoviocytes (FLS), the dominant non-immune cells of synovial tissues, mainly contribute to joint destruction via multiple mechanisms. In RA, FLS respond to endogenous ligands of pattern recognition receptors (PRRs) and inflammatory cytokines as non-immune cells. In addition, FLS aid in the activation of immune responses by interacting with immune cells and by supporting ectopic lymphoid-like structure (ELS) formation in synovial tissues. Moreover, FLS directly cause the pathogenicity of RA i.e., joint deformities. Here, we describe new findings and review the mechanisms underlying the regulation of immune reactions by non-immune FLS and their roles in inflammatory diseases such as RA.

## Introduction

Non-immune cells of target organs play essential roles in the pathogenesis of chronic inflammatory and autoimmune diseases, forming the basis of the unique features of each disease (1). Fibroblast-like synoviocytes (FLS) are non-immune cells found in synovial tissues. FLS function in the pathogenesis of rheumatoid arthritis (RA), a type of chronic systemic arthritis. Autoantibodies, such as rheumatoid factor (RF), and anti-citrullinated peptide antibodies (ACPAs) are unique features of RA, and their presence indicates strong involvement of CD4^+^ T cells and B cells in the RA pathogenesis (2). Therefore, cellular communication between FLS and hematopoietic immune cells may play a large role in the RA pathogenesis, including local autoantibody production in the RA synovium.

The synovium is a membranous organ lining the joint cavity. In normal physiological conditions within the joint cavity, the synovium supplies nutrients and the extracellular matrix (ECM) components of cartilage (3). FLS also strongly participate in the pathogenesis of RA. FLS support the development of the hyperplastic RA synovium as tertiary lymphoid organs (TLOs) by interacting with immune cells and organizing ectopic (tertiary) lymphoid-like structures (ELSs). Furthermore, FLS directly exert RA effector functions, which lead to joint deformity through osteoclastogenesis and the production of extracellular protease enzymes.

In this review, we describe new findings and examine the role of FLS in the RA pathogenesis.

## FLS Responses via PRRs

Like stromal cells in other organs, such as the skin, gingiva, and lymph nodes (LNs), FLS play a role as innate immune cells by recognizing invading pathogens via PRRs such as Toll-like receptors (TLRs). Among the human TLR family, TLR1-7 is expressed by FLS (4). TLRs can recognize components of both pathogens and endogenous factors. Double-stranded and single-stranded RNA are recognized by TLR3 and TLR7, respectively. Necrotic cells in inflamed joints may be a source of endogenous ligands for these receptors (5). Endogenous ligands, such as heat-shock proteins and low-molecular-weight hyaluronan, were initially reported to be directly recognized by a heterodimer of TLR2/TLR4. However, highly pure ligands do not activate these receptors (6).

Of note, citrullination of endogenous ligands, such as fibrinogen and histones, stimulates the TLR4-mediated pathway (7, 8). Anti-TLR4 antibody significantly blocks the activation of monocytes by synovial fluid from RA patients exhibiting ACPAs (9), suggesting involvement of the TLR4-mediated pathway in the pathogenesis of RA. FLS are not activated by TLR9 plus CpG DNA (10). However, neutrophil extracellular traps (NETs) are internalized via the receptor for advanced glycosylation end products (RAGE)–TLR9 pathway, followed by promotion of the FLS inflammatory phenotype and human leukocyte antigen (HLA) class II upregulation (11). Thus, FLS recognize both pathogens and endogenous ligands through the PRRs, and these interactions lead to the pathogenesis of RA.

## FLS Interactions With Immune Cells

Autoantibodies, such as RF and ACPAs, are an important feature of RA, and their presence provides evidence of the involvement of CD4^+^ helper T cells and B cells in the RA pathogenesis (2). The RA synovium frequently (40%) exhibits ELSs, which are discrete clusters of T cells, B cells, and macrophages (12). Consistent with the activated immune responses of these ELSs (1), B cells clonally expand in the RA synovium, presumably due to autoantigens, rather than in peripheral blood (13). During these activated responses, communication between FLS—a type of stromal cell—and immune cells may lead to the signature RA phenotype. In this section, we review the interactions of FLS with each cell type.

### Interactions With Macrophages

**Figure 1:**
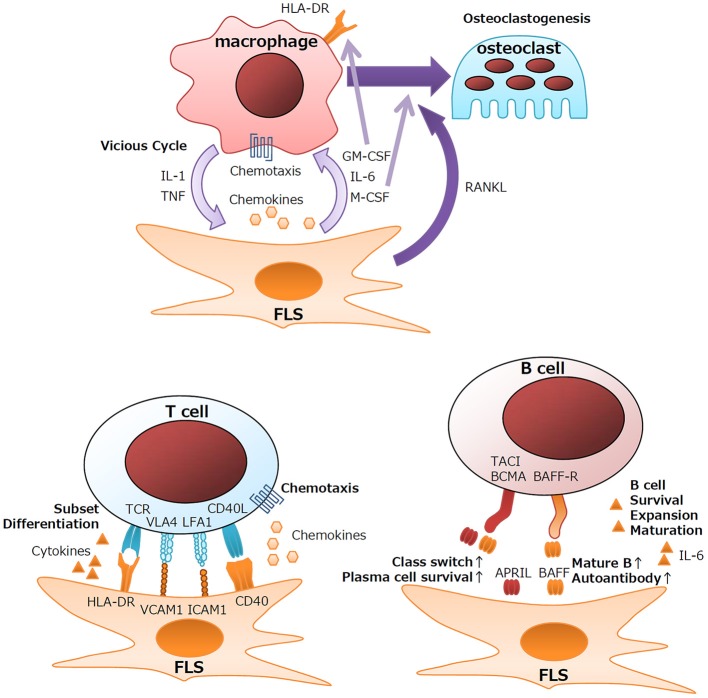
Schematic outline of interactions between FLS and immune cells.

Under healthy conditions, resident monocytes are found in the intimal lining and sublining of synovial tissues (3, 14). Upon activation of synovial tissues, neoangiogenesis and chemokine recruitment function in the influx of peripheral monocytes into the synovium (3). In response to proinflammatory cytokines, FLS secrete chemoattractants, such as CCL2, CCL5, CCL8, CXCL5, and CXCL10, which leads to the recruitment of monocytes and macrophages (3). Cytokine networks at inflammatory sites contribute largely to the RA pathogenesis and the perpetuation of inflammation. Detailed analysis of the cytokine milieu of RA synovitis revealed that macrophages and fibroblasts are the major sources of proinflammatory cytokines (15). Anti-cytokine therapies, including anti-TNF and anti-IL-6, markedly improve the clinical results after RA treatment (16, 17). These cytokines form a vicious inflammatory cycle leading to synovial hyperplasia, influx of lymphocytes, and the production of effector proteins. Macrophages are the major source of IL-1β and TNF, and FLS in the intimal lining are the main source of IL-6 (15). Colony-stimulating factors, such as GM-CSF and M-CSF, are also produced primarily by FLS in the intimal lining (18). Upregulated GM-CSF production by IL-1β/TNF-stimulated FLS is involved in the local expansion of macrophages. GM-CSF rather than IFN-γ plays an important role in the induction of HLA class II expression on macrophages in the RA synovium (Figure 1) (15). Indeed, anti-CXCL10 treatment and anti-GM-CSF receptor treatment are clinically effective for RA (19, 20).

Another aspect of the interaction between macrophages and FLS is the induction of osteoclasts, which are specialized bone-absorbing cells that differentiate from macrophages. Actively transformed RA synovium, the so-called pannus, destroys the cartilage matrix and can invade bone. At the tip of the pannus, multinuclear cell osteoclasts greatly absorb adjacent bone. RANKL has been identified as the factor responsible for the differentiation of osteoclasts from macrophages (21–23). Activated FLS produce large amounts of RANKL and another essential factor, M-CSF. Clinically, anti-RANKL antibody significantly attenuates the bone destruction of RA (24).

### Interactions With T Cells

CD4^+^ helper T cells are another important player in the RA pathogenesis. Genetic studies of RA-related genes revealed that T-cell-related genes, including *HLA-DR, PTPN22*, and *CTLA4*, are involved in RA (25), and that treatment that targets T cells is as effective as anti-TNF therapy (26). CD4^+^ T cells differentiate into several types of subsets depending on the differentiation environment. IL-17-producing helper T (Th17) cells, follicular helper T (Tfh) cells, and PD-1^hi^CXCR5^−^ peripheral helper T (Tph) cells are thought to be involved in RA (27–29). Th17 cells function in the activation of FLS, macrophages, endothelial cells, and chondrocytes mainly via the biological effects of IL-17A (30). However, clinical trials of neutralizing anti-IL-17 antibodies demonstrated that Th17 cells play a role in the pathogenesis of psoriatic arthritis but less in that of RA (31, 32). The strong involvement of autoantibodies, such as RF and ACPAs, in RA suggests that B-helper activity is a key function of CD4^+^ helper T cells. In LNs or tonsils, Tfh cells exert B-helper activity, and aid in class switching and affinity maturation of antibodies via the activity of the master transcription factor BCL-6 (33). However, the BCL-6 expression level is not increased in RA synovial CD4^+^ T cells (34, 35) despite production of autoantibodies in the RA synovium (13). Recently identified by comprehensive analysis of clinical samples as a pathogenic CD4^+^ subset in RA patients (36), Tph cells also play a role in B-helper activity and ELS formation at inflammatory sites (34, 35, 37).

Although not primary immune cells, FLS express immune-related genes, including HLA Class II, the gene required for presenting antigens to CD4^+^ helper T cells, during the development of RA. FLS also function in the differentiation of T cells via cytokine production. TGF-β is known to be involved in the differentiation of several types of T-cell subsets, such as inducible T reg (iTreg), Th17, and Tph cells (38), by inducing the transcription factors FoxP3 (39), RORC (40), and Sox4/Maf (37). Chemokines from FLS also help recruit T cells. CXCL9/10/11, CCL20, and CCL2 recruit Th1, Th17, and Tph cells via the cytokine receptors CXCR3, CCR6, and CCR2, respectively (36, 41–43). RA FLS also significantly express higher amounts of CX3CL1 (fractalkine), and the expression of its sole receptor, CX3CR1, is upregulated in CD4^+^ and CD8^+^ T cells of patients with RA, suggesting the involvement of the CX3CL1/CX3CR1 axis in the pathogenesis of RA (44). Consistent with this, anti-CX3CL1 treatment has significant clinical effects for RA (45). Membrane proteins and adhesion molecules also lead to the activation of T cells and FLS. CD40L produced by CD4^+^ T cells stimulates B-cell activity by stimulating CD40 signaling in B cells. Similarly, CD40L produced by T cells also stimulates FLS to release chemotactic molecules (46). LFA-3 on FLS and LFA-2 (CD2) on T cells are important for strengthening the adhesion between T cells and FLS (47). ICAM-1 and VCAM-1 expressed on FLS regulate the development of T cells by interacting with the integrins LFA-1 and VLA-4, respectively (48). Thus, FLS support the immunological functions of T cells via pleiotropic mechanisms (Figure 1).

### Interactions With B Cells

The clinical relevance of autoantibodies in RA supports the important roles of B cells in the RA pathogenesis. Indeed, administration of the B-cell-depleting anti-CD20 antibody, rituximab, produces good clinical results for RA (49). Autoantibodies develop initially in the synovium rather than in peripheral blood and are class-switched during the development of RA (13), which indicates that the local synovial environment is a main contributor to the development and maturation of autoantibody-producing B cells.

Upon TLR3 stimulation, FLS produce large quantities of B-cell-activating factor (BAFF) and a proliferation-inducing ligand (APRIL) (50). Although both BAFF and APRIL bind to the receptor's B-cell maturation antigen (BCMA), transmembrane activator, and cyclophilin ligand interactor (TACI), only BAFF can bind to the BAFF receptor (BAFF-R) (51). Therefore, BAFF and APRIL exert different biological effects on B cells. BAFF is important for the maturation and survival of B cells; upregulated BAFF expression leads to an increase in the number of mature B cells and autoantibody production in mice (52). In contrast, APRIL plays essential roles in class-switching of antibodies and the survival of plasma cells (51). However, the roles of BAFF and APRIL in RA remain to be determined. Anti-BAFF treatment for RA downregulates RF but has little effect on the clinical course of disease activity (53).

IL-6 is another key factor secreted by FLS that can affect B cell functions. In addition to its pleiotropic effects on multiple cell lineages, IL-6 plays key roles in the development of B cells. IL-6 is involved in the survival, expansion, and maturation of B cells (54), and it functions in the commitment to the Tfh cell subset via the induction of the transcription factor BCL-6 (55). Therefore, FLS support the function of B cells, including autoantibody production, and lead to the pathology of RA (Figure 1).

### FLS Support ELS Formation

**Figure 2:**
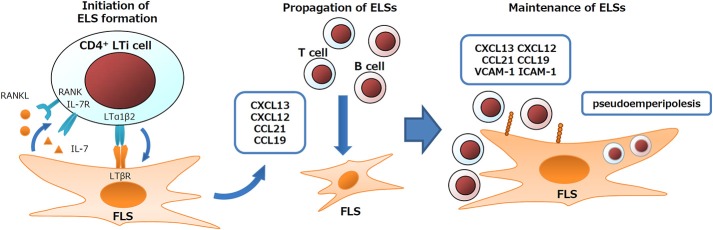
Schematic outline of FLS contribution to TLO formation.

Frequent ELS formation is an important feature of the RA synovium (12). In ELS, generally upregulated immune responses lead to autoantibody production in rheumatic diseases, or to anti-viral immunity or anti-tumor immunity depending on the features of diseases (1, 56–58). FLS play essential roles in ELS formation and in the regulation of ELS immune responses. During the developmental process of secondary lymphoid organs, such as LNs and tonsils, interactions between lymphoid tissue inducer (LTi) cells and lymphoid tissue organizer (LTo) cells coordinate organ formation. Similarly, FLS express signature genes of LTo cells and their derivative fibroblastic reticular cells (FRC) such as LTβR, IL-7, RANKL, CXCL13, CXCL12, CXCL21, CXCL19, VCAM-1, ICAM-1, and gp38 (12, 34, 48, 59–64). In addition, FLS also exert nurse-like activity by supporting lymphocyte pseudoemperipolesis (active migration of lymphocytes into the cytoplasm of nurse-like cells). FLS under RA or of non-RA conditions support the pseudoemperipolesis of T cells, B cells, and NK cells, aiding in their survival, activation, or functions such as IgG production (60, 65, 66). Due to such activity of FLS, TLO cells are (i) induced via initiators, such as LTαβ, IL-7, and RANKL (67), (ii) expanded via propagators, such as CXCL13/12/21/19, and (iii) maintained via adhesion molecules and nurse-like activity (Figure 2).

### Involvement in Organ-Specific Immune Responses

Although ELS exhibit an overlapping structure in several inflammatory diseases, the target autoantigens depend on the diseases. In the salivary gland ELSs of patients with Sjögren syndrome, B cells and plasma cells are frequently reactive against the ribonucleoproteins Ro/SSA and La/SSB, whereas autoantibodies specific for RA are RF and ACPA. These differences may be partly attributed to the differences in non-immune cells of target organs. One notable feature of FLS is their contribution to the joint architecture via formation of synovial anatomical components: the intimal lining and sublining. Indeed, FLS grown in three-dimensional culture self-direct their architecture to be very similar to that of the intimal lining (14), which borders between joint spaces and synovial tissues. This signature architecture of joints may function in the enrichment of disease-specific antigens, such as synovial fluid NETs, whose citrullinated histones are major targets of ACPA (12, 68, 69). Alternatively, a protein of FLS, citrullinated calreticulin of FLS, or citrullinated aggrecan from cartilage are other targets for RA autoreactive B cells or T cells, respectively (70, 71). These findings suggest a connection between autoantibodies and organ-specific antigens depending its structure and components.

## FLS Exert Effector Functions of RA

As a consequence of activated immune responses, hyperplastic synovial tissues of RA (pannus) aggressively invade adjacent cartilage, tendon, and bones, leading to the destruction of multiple joints. Clinical studies demonstrated that the vasculature of the RA synovium reflects joint inflammation and correlates with future joint deformities. In this joint destruction, FLS directly exert effector functions. In this section, we discuss FLS effector functions and their regulation.

### Synovial Hyperplasia

As mentioned above, FLS are highly involved in the formation of the intimal lining of synovial tissues (14). The intimal lining comprises 1–3 cell layers in normal physiological conditions, but its thickness increases to 10–15 cell layers in the activated RA synovium (3, 46). Upregulated expression of effector factors in the hyperplastic intimal lining suggests the importance of the dysregulation of the intimal lining in the RA pathogenesis (15, 18). Dysregulation of apoptosis and proliferation of FLS via multiple genes may play a role in this hyperplasia (72–74). In three-dimensional culture conditions, the combination of growth factors (e.g., PDGF and TGF-β) with inflammatory cytokines (e.g., TNF) strongly induces hyperplasia of the synovial lining via activation of the PI3K–Akt pathway (75, 76). Adhesion molecules, such as cadherins and integrins, play essential roles in the formation and maintenance of the synovial lining. A deficiency in cadherin 11 leads to the disappearance of the intimal lining in mice (77). Integrin α9β1 is also preferentially expressed by FLS. Neutralization of integrin α9β1 or knockdown of its ligand tenascin-C abrogates the formation of the synovial lining (78). Based on these findings, in addition to integrin α9β1 and cadherin 11, FLS may play a role in the formation of the synovial lining. These adhesion molecules also have effector functions in RA. RA FLS cultured in a three-dimensional manner secrete greater amounts of effector factors, such as MMP1, MMP3, IL-6, or RANKL, than monolayer FLS.

The protein gp38, a FRC signature gene, may also function in the regulation of TLO size. Regarding LNs, gp38 is involved in the FRC regulation of LN size. Interaction of gp38 with its ligand CLEC2, which is preferentially expressed by dendritic cells in LNs, reduces the tension of fibroblastic reticular cells. Enlargement of LNs upon inflammation is significantly disturbed in gp38-deficient mice (79). Of note, RA FLS of the intimal lining express more gp38 than OA FLS (62), and platelets in the synovium preferentially express CLEC2 (80). However, it remains to be investigated whether gp38 is also involved in hypertrophy of the RA synovium. Treatments targeting integrins or other adhesion molecules may be candidate alternatives for patients with refractory synovial hyperplasia.

### Neoangiogenesis in the RA Synovium

The transitory pre-vascular inflammatory stage of the RA synovium is followed by a prominent vascular stage (46, 81), which is clinically detectable by power Doppler (PD) sonography as a reliable sign of active synovitis, and is significantly correlated with the poor prognosis of RA (82). The clinical connection between PD-positive synovial hyperplasia and the poor prognosis of RA strongly suggests the importance of the synovial vasculature in the RA pathogenesis. Hyperplasia of the intimal lining and infiltration of T cells, B cells, and macrophages into the sublining increase the metabolite demand and hypoxia, which induces marked new vessel formation (81). In particular, hypoxia accompanied by synovial hyperplasia drives the production of VEGF, the most important factor for neoangiogenesis, via the hypoxia-inducible transcription factor (HIF)-1α, whereas HIF-2α is involved in FLS functions of intimal lining (83–86). Subsequently, upregulated VEGF leads to the activation of the angiopoietin (Ang)/Tie-2 system. Although Ang1 is constitutively expressed by quiescent vasculature, the expression of Ang2 depends on endothelial cell activation (87). The activation of Tie-2 signaling via the Akt pathway is required for the proliferation and survival of endothelial cells (81). Observation of Tie-2 activation in synovial tissues from some unestablished RA patients might imply the involvement of angiogenesis process in the development of RA (88). Of note, RA FLS under hypoxic conditions are sufficient for angiogenesis employing multiple factors such VEGF, bFGF, TGF-β, IL-6, IL-8, CXCL12, ICAM-1, VCAM-1, and matrix metalloproteinases (84, 89). In the context of the clinical relevance of PD for the development of joint destruction, treatments targeting HIF-1α or angiogenic factors have been discussed as alternative treatments for RA (81, 90).

### Direct Effector Functions of FLS

One important feature of RA is the direct contribution of FLS to the degeneration of joints. Models of FLS transplanted together with cartilage into immunodeficient mice demonstrated that once activated, RA FLS acquire an aggressive phenotype that invades adjacent cartilage (91). RA FLS secrete multiple species of extracellular protease enzymes such as matrix metalloproteinases (MMPs). MMPs can be subdivided according to their substrates into collagenases, stromelysins, gelatinases, and membrane-type MMPs. The collagenases MMP-1 and MMP-13, and the stromelysin MMP-3 are the most important MMPs in the RA pathogenesis (3). FLS also produce tissue inhibitors of metalloproteinases (TIMPs). Cartilage destruction depends on the balance between MMPs and TIMPs. When the balance favors MMPs, cartilage degradation proceeds. The expression of MMPs, but not TIMPS, is upregulated by inflammatory cytokines (IL-1β, TNF, and IL-17) (3), which is consistent with the correlation between inflammation and cartilage degradation.

A disintegrin and metalloproteinase with thrombospondin motifs (ADAMTs) comprise another family of extracellular proteases. ADAMTS4 and ADAMTS5 produced by FLS lead to cartilage damage in RA (92). Adhesion molecules, such as integrins and cadherins, are also involved in cartilage degeneration. As described above, FLS in the intimal lining increase the expression of MMPs via signaling of integrin α9 and cadherin 11 (77, 78). RA FLS also express higher amounts of integrin α5β1, which plays an important role together with syndecan 4 in the adhesion of cells to, and destruction of, the cartilage matrix (93). Another essential function of FLS is osteoclastogenesis, which is also involved in joint destruction, via RANKL and M-CSF secretion. Although activated T cells also produce RANKL, conditional knockout *in vivo* experiments revealed that FLS contribute to bone destruction more than T cells (63).

## Conclusion

Several studies on RA have confirmed that FLS—non-immune cells found in target organs—play several roles in disease development. These findings have increased our understanding of the immune responses of ELSs at local inflammatory sites. However, many questions remain to be answered about the immune responses at local inflammatory sites, including autoantibody development in ELSs and the complex roles of helper T cells. Recent single-cell analysis has demonstrated that FLS can be classified into several subsets (94). Further investigation of the interactions between FLS and immune cells will improve our understanding of human immunology and aid in the development of new RA treatments.

## Author Contributions

The author confirms being the sole contributor of this work and has approved it for publication.

### Conflict of Interest Statement

The author declares that the research was conducted in the absence of any commercial or financial relationships that could be construed as a potential conflict of interest.
